# Editorial: Intrinsic capacity and resilience vs. frailty: On the way to healthy aging

**DOI:** 10.3389/fmed.2023.1155648

**Published:** 2023-02-21

**Authors:** Elena Frolova, Beatrice Arosio, Wee Shiong Lim

**Affiliations:** ^1^The Family Medicine Department, North Western State Medical University, St-Petersburg, Russia; ^2^Department of Clinical Sciences and Community Health, University of Milan, Milan, Italy; ^3^Department of Geriatric Medicine, Institute of Geriatrics and Active Aging, Tan Tock Seng Hospital, Singapore, Singapore; ^4^Lee Kong Chian School of Medicine, Nanyang Technological University, Singapore, Singapore

**Keywords:** intrinsic capacity, frailty, healthy aging, biomarkers, psychosocial aspects, geroprotective, interventions, sarcopenia

One of the most intriguing aspects of aging is the heterogeneity of the older adult population. Some age rapidly and lose their independence, while others remain physically active and cognitively preserved despite their age and number of comorbidities (1). Although geriatricians have long used the concept of frailty as a measure of an individual's risk profile in clinical practice, there is increasing appreciation that the unitary concept of frailty may not adequately address all situations.

The World Health Organization (WHO) advocates moving away from a disease-focused model of aging and frailty, and toward a more positive model of healthy aging (2) that focuses on preserving functional ability through optimizing intrinsic capacity (IC) and the environment. IC is defined as “the composite of all the physical and mental capacities that an individual can draw on” at any point in time (3). IC includes five domains, namely locomotion, vitality, sensory, cognition, and psychological (4–6). These domains influence each other and are, in turn, influenced by environmental factors (7). The related concept of physical resilience has been defined as one's ability to resist decline or recover from functional decline after a significant health stressor (8).

However, to date, the tools that geriatricians can use to measure the contribution of each domain to the IC model, as well as the factors and mechanisms that contribute to sustainability and physical reserve, are not fully understood. This highlights the need for more studies to understand the inter-dependent yet distinct contribution of IC and resilience vis-à-vis frailty toward healthy aging (3, 9). This Research Topic is therefore timely, with the over-arching goal of bridging the knowledge gap about the healthy aging model. We aim to demonstrate the possibility of interventions to return the aging processes from pathological to healthy, describe the difference between frailty and IC, and explicate the mechanisms of resilience and physical and cognitive reserves.

Notwithstanding the consensus regarding the concept of frailty aligning with either the phenotypic or deficit accumulation model, debate persists about the tools for assessing and measuring frailty in clinical practice (10). Of note, it is imperative to consider the national, cultural and organizational context in which screening tools are administered. In this regard, Jung, Baek, Kwon, et al. describe their experience using the Clinical Frailty Scale (CFS) to evaluate patients in the emergency department of a busy Asian hospital. They demonstrated that CFS administered in the emergency department could predict adverse events, such as the development of pressure sores, delirium, falls, repeated hospitalizations, and placement in long-term care institutions. Thus, the choice of a tool for screening and measuring frailty is determined largely by the clinical purpose of frailty identification. Another article by Jung, Baek, Jang, et al. compared the original classification and culturally modified classification of the CFS by considering the culturally-sensitive items of food preparation and household chores in instrumental activities of daily living (IADL) which may be less applicable to older men in Korea. The main implication is the reclassification of CFS 5 (impairment in IADL) to CFS 4 in the affected men. The results demonstrate that the modified CFS had better construct validity and comparable predictive validity for the composite outcome of institutionalization and death, alluding to the salience of cultural adaptation of selected items for accurate frailty assessment in older persons. In addition, it is important to consider the perception of older persons toward their functional capabilities, especially in the context of concomitant cognitive impairment (11). The paper by Hartle et al. highlights the lack of awareness of ADL in persons with dementia, and the relevance of informant reports and cognitive testing for fluency to complement clinical assessment of ADL performance. Regarding awareness, general cognitive level was a significant predictor of instrumental ADL awareness, and memory was the only predictor of awareness of basic ADL.

In recent years, there is increasing interest within the field to understand the biological basis of IC and its component domains, in particular, the vitality domain (12–15). Four papers in this Research Topic shed further insights into possible mechanisms and pathophysiology which underpins the biological basis of IC. Meng et al. set out to justify not only the assessment of each domain, but also the overall composite assessment of the IC, in association with the functional status. In addition, they tried to ascertain the biological basis of IC and determine the prognostic value of this estimate for 4-year mortality. The results showed that a scoring system considering different weights of individual IC subdomains not only predicts mortality but also suggests different pathophysiologies across the life course of aging, including inflammation, nutrition, stress, and the ApoE4 genotype. The remaining three papers examine the important entity of sarcopenia, which predisposes to adverse outcomes such as reduced mobility, functional decline, falls, institutionalization and mortality, and has been proposed to be the antecedent of physical frailty (16, 17). Lu et al. investigated the association of sarcopenia with the fasting insulin level and other markers of lipid and insulin metabolism in both diabetic and diabetes-free older persons. They reported that sarcopenia is associated with low insulin levels, regardless of diabetic status, and also uncover interesting associations with leptin, C-peptide, and Insulin Growth Factor-1. In their Perspective article, Chew et al. explore the recent experimental and clinical evidence in support of the novel interaction between gut microbiota and muscle function in the gut-muscle crosstalk and discuss potential exercise and pharmacological interventions which may influence the microbiome to provide novel approaches to the treatment of sarcopenia and frailty. Another area of emerging interest is to understand the relationship between chronic diseases with IC and vitality in order to accrue fresh insights for early intervention (18). Loh et al. provided a comprehensive commentary of the cardio-sarcopenia syndrome which refers to the co-occurrence of alterations in myocardial structure in older adults with skeletal muscle sarcopenia. Investigations into this syndrome have spurred a fresh level of interest in the cardiac-skeletal muscle axis and raise the tantalizing possibility of inter-disciplinary interventions aimed at improving the condition of skeletal muscles, such as resistance exercises, aerobic physical activity and dietary protein consumption, to improve myocardial function.

Three articles in the Research Topic touch upon the psychosocial aspects of IC. The cognitive and psycho-emotional domains of IC are determined not only by the individual characteristics of each person, but also by the state of one's social environment (19, 20). Fang et al. evaluated the social support of frail, pre-frail and robust elderly and showed that the frail and pre-frail have a lower level of social support than the robust. Chen et al. reported in their cross-sectional study of 3 cities in China that moderate-to-strong levels of sense of coherence conferred lower odds of being frail and proposed improving sense of coherence as a possible strategy to prevent frailty. Lastly, using latent cluster analysis, Merchant et al. identified three patterns of participation restriction (low/moderate/high) in community dwelling older adults ≥60 years with falls or risk of falls. Of note, the presence of 3 out of 6 impaired IC denotes a >80% probability of belonging to the low/moderate participation class. The identified IC risk factors of physical functioning, cognitive status, hearing impairment and malnutrition may thus be potential intervention targets to improve participation of older adults with falls or at risk of falls.

The next major theme in our Research Topic revolves around the area of interventions to regress the process of pathological aging and to restore healthy aging. In the area of outcome measures of multidimensional aging, Zhang et al. developed a new composite measure of aging that integrated phenotypic and functional aging with potential for assessment of geroprotective programs. This composite measure better predicted mortality risk compared with each aging measure in isolation, and was responsive to modifiable lifestyle factors including smoking status, body mass index, alcohol consumption, and leisure-time physical activity. Tan et al. demonstrated preliminary evidence of a novel technology-enabled autonomous multi-domain community-based interventions with exercise, nutrition, and polypharmacy components in improving frailty status, physical performance and strength in pre-frail older adults. Using the Senior Technology Acceptance and Adoption Model, the study also explicated user experience insights which can affect the usability and enjoyment of technological interventions for older persons. In their non-randomized controlled study of a multi-domain exercise and nutrition intervention in pre-frail older persons, Tay et al. reported that reversal to robustness at 1-year was similar between intervention and control groups, suggesting that focusing only on the locomotion and vitality domains may not adequately address component domain losses to optimize pre-frailty reversal. Furthermore, the intriguing result that IC rather than intervention exposure influences reversal to robustness suggest that an IC-guided approach to target identified domain declines may be more effective in preventing frailty progression. Lastly, in the systematic review of multi-domain and lifestyle interventions to support IC, Bevilacqua et al. reported that the majority of successful interventions are based on a goal setting approach whereby older persons are actively and involved in defining the intervention goals. The observation that there were no studies which utilized the IC framework to design the intervention, highlights a significant gap which can inform the future research agenda.

As guest editors, the 14 papers presented in this Research Topic provide a valuable compendium of fresh insights and perspectives in the rapidly-growing field of IC, frailty, and healthy aging. It is our sincere hope that this Research Topic will spur further conversations and explorations to advance this exciting field of research in geroscience and geriatrics.

## Author contributions

EF, BA, and WL contributed to conception of the editorial. EF wrote the first draft of the manuscript. All authors contributed to manuscript revision, read, and approved the submitted version.
